# Association Between Mild Cognitive Impairment and Seasonal Rest-Activity Patterns of Older Adults

**DOI:** 10.3389/fdgth.2022.809370

**Published:** 2022-02-23

**Authors:** Christina Reynolds, Nora Mattek, Miranda M. Lim, Zachary Beattie, Hiroko H. Dodge, Jeffrey Kaye

**Affiliations:** ^1^Department of Neurology, Oregon Health and Science University, Portland, OR, United States; ^2^Oregon Center for Aging and Technology, Oregon Health and Science University, Portland, OR, United States; ^3^National Institute on Aging (NIA)-Layton Aging and Alzheimer's Disease Center, Portland, OR, United States; ^4^Veterans Affairs (VA) Portland Health Care System, Portland, OR, United States

**Keywords:** aging, sleep, circadian rhythms, mild cognitive impairment, in-home monitoring

## Abstract

Seasonal variation in rest-activity patterns has been observed in healthy adult populations. This study examined seasonal variation in total time spent overnight in the bedroom by cognitively intact older adults and older adults with mild cognitive impairment (MCI). We hypothesize that seasonal variation in rest-activity patterns is observed in the cognitively intact group and that this variation is disturbed in those with MCI. Study participants were 128 older adults; mean age 85.2 years. Ninety-eight were cognitively intact, and 30 had been diagnosed with MCI. All were enrolled in an ongoing longitudinal study using in-home passive monitoring technology. Infrared presence sensors were placed throughout each participant's home to monitor movement and presence in each room of the home. Activity data was collected from the sensors over a period of up to 527 days. Overnight time in bedroom was found to vary seasonally for the cognitively intact group, with longer times spent overnight in the bedroom during the winter months. This seasonal variation was not observed for those with non-amnestic MCI. MCI is associated with an attenuation of seasonal variation in total time spent in the bedroom at night. Detection of changes in infradian sleep patterns may be an early marker of cognitive decline. Which key determinants are driving these disturbed rhythms, such as features intrinsic to changes in the brain or to environmental factors or external cues, remains an important question for ongoing and future studies.

## Introduction

Normal aging is associated with many changes in sleep patterns such as increasing sleep fragmentation, insomnia, and difficulty falling asleep (1). Sleep quality is closely linked to cognitive function with poor sleep quality affecting cognitive performance even in healthy aging adults (2). This is of further concern because poor sleep quality is associated with a variety of health concerns, ranging from an increase in falls to cognitive changes and depression (3). Inadequate sleep has been shown to be correlated with increasing amyloid beta concentrations which may culminate in Alzheimer's disease (4). Mild cognitive impairment (MCI), a clinical precursor to Alzheimer's disease is associated with altered sleep patterns, including increased wake after sleep onset and sleep latency (5, 6). These sleep changes may provide a way of identifying older adults who are experiencing early changes leading to dementia. Thus, monitoring sleep offers a valuable window into the ongoing cognitive status of the older population.

It is well established that sleep habits are strongly influenced by circadian rhythms on a daily scale (7). Sleep patterns also vary seasonally with change in the length of the day with longer sleep periods and more sedentary behavior observed during winter months (8–12). For healthy adults, this seasonal effect on sleep duration may be related to individual chronotype, with those tending to be more active in the evening more affected by seasonal changes (13). A longitudinal study followed the sleep patterns of 216 adults across the United States for 1 year, also finding that wake times and sleep duration varied with seasons with wake times becoming earlier in the spring (14). These natural sleep patterns are affected by aging, though it is not well understood why. It is possible that circadian regulation of sleep weakens with age (15), resulting in less consolidated sleep. Sensitivity to zeitgebers, or other environmental cues, may also be lessened by aging. Older adults have been shown to be less responsive to light exposure (16), which may be a result of the yellowing of the lens of the eye which occurs with aging (17). Disrupted nightly sleep has been documented in several studies of mild cognitive impairment (MCI) which occurs with increasing frequency with age (5, 18, 19). While changes in sleep patterns have been observed with seasons, studies of cognitive function in persons with Alzheimer's disease did not show a seasonal effect (20).

The objective of this study was to examine the rest-activity patterns habits of older people with and without MCI over a longer time scale than has been previously studied. This longitudinal assessment provides the opportunity to determine whether normal seasonal (infradian) rhythms are disrupted in addition to the disruption of circadian rhythms already observed with aging. We hypothesized that fewer hours of total time spent overnight in the bedroom would be observed in the summer for the cognitively intact group and that this seasonal rest pattern would be disturbed in the group with MCI. Our study draws on a cohort of older individuals who have had passive infrared activity sensors unobtrusively deployed in their home for up to 18 months to continuously assess natural activity patterns in individuals with and without MCI in their familiar home environment.

## Methods

### Subjects

One hundred and twenty-eight ambulatory community-dwelling volunteers (mean age 85.2 ± 7.7 years, 100 female) were included in this study. All participants provided written informed consent and were enrolled in one of two ongoing studies of in-home monitoring: the ORCATECH Life Laboratory study and the Intelligent Systems for Assessing Aging Changes (ISAAC) study (21). The study protocols were approved by the Oregon Health and Science University Institutional Review Board (Life Laboratory, IRB #2765; ISAAC IRB #2353). Participants were recruited from the Portland, Oregon metropolitan area through advertisements and presentations at local retirement communities. Inclusion criteria were ≥ 60 years of age for the Life Laboratory and ≥ 80 years for ISAAC. Participants had to be living alone independently and were required to not have a dementia diagnosis with a Mini-Mental State Examination (MMSE) (22) score > 24 and a Clinical Dementia Rating (CDR) (23) ≤ 0.5. Exclusion criteria included illnesses that would hinder long-term daily follow-up, such as end-stage cancer. All participants diagnosed with mild cognitive impairment were classified as having MCI (amnestic or non-amnestic) using the Jak et al. criteria (24). All participants had an annual standard battery of clinical and neuropsychological assessments including the MMSE, CDR, Geriatric Depression Scale (25), Functional Activities Questionnaire (26), and Cumulative Illness Rating Scale (27) (Table 1).

**Table 1:** Demographic and clinical characteristics by cognitive group (*n* = 119).

	**Intact (*n* = 98)**	**Non-amnestic MCI (*n* = 21) (standard deviation)**	***p*-value**
Age	84.3 (7.4)	87.9 (8.5)	0.06
Sex (% female)	80%	86%	0.76
Education, years	15.9 (2.5)	14.2 (2.9)	0.01
MMSE	29.1 (1.1)	26.8 (2.7)	<0.0001
CIRS	20.3 (2.5)	21.3 (2.8)	0.03
FAQ	0.5 (1.9)	1.4 (2.5)	0.17
GDS	0.9 (1.2)	2.0 (3.3)	0.12
BMI	26.8 (4.6)	28.7 (5.0)	0.11
Days of available sleep data	527 (152)	465 (195)	0.26

*A comparison of the demographic information describing the cognitively intact study group and the group of participants with non-amnestic mild cognitive impairment*.

*MMSE, Mini-Mental State Examination; CIRS, Cumulative Illness Rating Scale; FAQ, Functional Activities Questionnaire; GDS, Geriatric Depression Scale; BMI, Body mass index*.

Nine volunteers (7%) were classified as having amnestic MCI using the Jak et al. criteria, 21 (16%) had non-amnestic MCI, and the remaining 98 (77%) were cognitively intact. Demographic and clinical characteristics across the group are shown in Table 1.

### Rest and Activity Monitoring

Participants' homes were instrumented using passive infrared presence sensors (NYCE, Inc.; Vancouver, BC). Homes included in the ORCATECH study range from one-bedroom apartments to homes with up to five bedrooms. Frequently visited rooms (bedrooms, bathrooms, kitchens, and living rooms), were outfitted with passive sensors (21). The sensors fire when presence activity is detected and a timestamp of each sensor firing is sent wirelessly to a transceiver. These sensors have a refractory period of 5 s. The data is then stored in a SQL database. An algorithm was written in MATLAB to estimate total rest time from the firing timestamps of the bedroom sensors and sensors in neighboring rooms. The total rest time estimated from this algorithm was validated previously using pressure mats (28).

### Statistical Analysis

Data consisted of three sets of longitudinal home-based activity time-series, divided into rest period measurements of cognitively intact individuals, individuals with amnestic MCI, and those with non-amnestic MCI. Total time spent in the bedroom at night was estimated for 128 individuals between February 27, 2015 and July 7, 2017. There was not a total time in bedroom value for every individual for each night in that time period, as individuals may have joined or left the study at differing times or data may be missing for some individuals for certain nights due to those individuals being out of the home. For each week in the study, the median of all total times in bedroom between 6:00 p.m. and 11:00 a.m. for each twenty-four-long period was estimated for all individuals was found. The median of the data was chosen because the median provides a better estimation of a typical value in the data in the possibility the data is skewed. We are estimated rest periods using an indirect method which may cause a skew in the data as it is possible to overestimate rest periods because the sensors cannot differentiate sedentary behavior from sleep.

Data was binned weekly to average out the effect of rest-activity patterns differing throughout the week, such as sleeping in on weekends. Because of the small sample size of the amnestic MCI group, there were weeks with as few as three individuals were represented in the calculation of the median total time in bedroom at night. For this reason, we did not to carry out the seasonal variation analysis on this group.

Data were analyzed to test for seasonal variability in two ways: application of cosinor analysis (29) and use of a Spearman correlation between the total time in bedroom at night and the number of nighttime hours.

**Figure 1:**
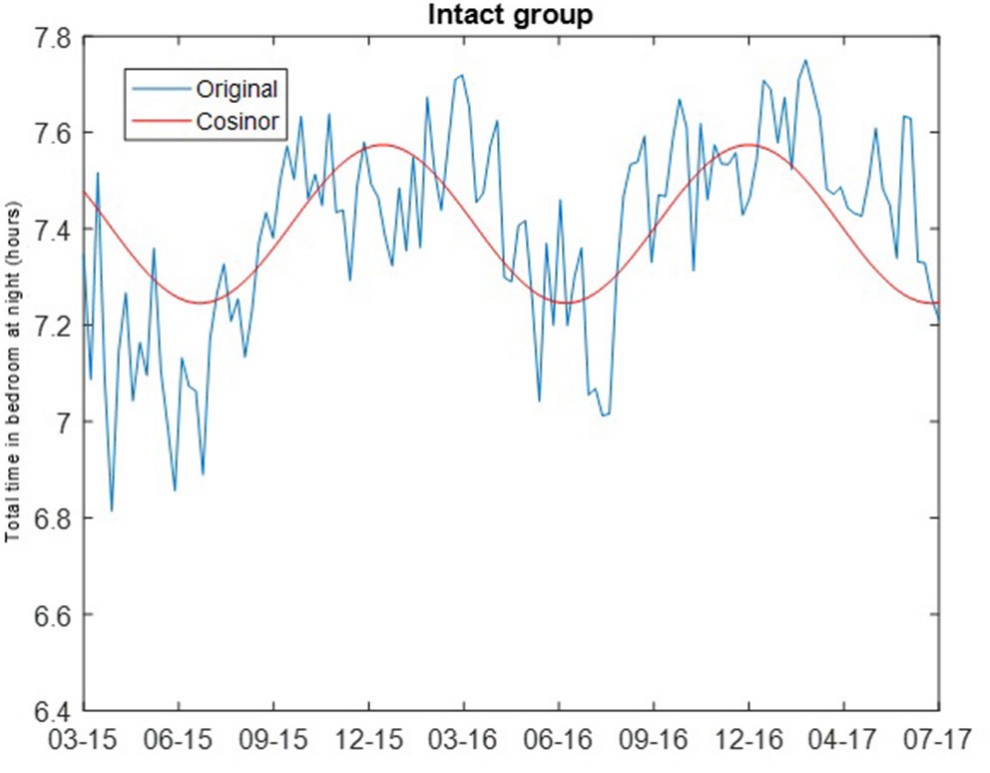
Cosinor fits of total time in bedroom at night of cognitively intact group. Cosinor fit for the cognitively intact group which shows a stronger relationship between total time in bedroom at night and day of year.

**Figure 2:**
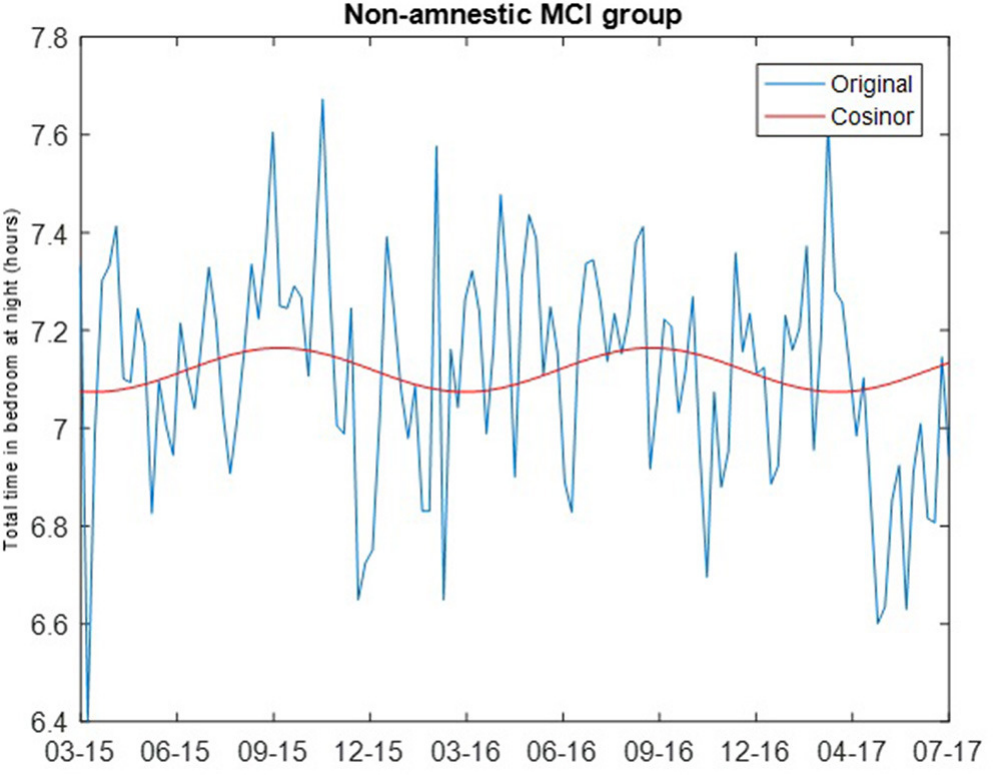
Cosinor fit of total time in bedroom of non-amnestic MCI group, showing a weaker relationship between total time in bedroom at night and day of year as seen in the intact group.

Single-component cosinor analysis is the application of a regression model for a single component written as:


(1) $$\begin{array}{r} Y\left(t\right)=M+A\;\cos\left(\frac{2\pi t}{\tau }+\varphi \right)+e\left(t\right)\; \end{array}$$

where M is the MESOR (Midline Statistic of Rhythm), A is the amplitude (a measure of half of the extent of variation within a cycle), ϕ is the acrophase (a measure of the time of the high value in each cycle), τ is the period, and e(t) is the error term (30). The least squares method is used to fit the observed data with the model. Cosinor analysis was carried out using the MATLAB function *cosinor* (31). For both the intact and non-amnestic MCI groups, the cosinor fit was carried out assuming a period of 365.24 days. Chronograms of the weekly median total sleep time for the cognitively intact and non-amnestic groups were plotted to visualize the data (**Figures 1, 2**).

The cosinor analysis was carried out for each individual, resulting in a value for amplitude, acrophase, and MESOR for each person's rest-activity pattern data. A multivariate logistic regression was carried out with the cosinor amplitude as the output and MCI status as the predictor. The MATLAB function *cosinor* was used to compute the rhythm detection test. The rhythm detection test, also called the zero amplitude test, tests the overall significance of the cosinor model fit to a data set. The rhythm detection test produces a *p*-value; a low *p*-value for the rhythm detection test signifies a high probability that that data is periodic. We chose a *p*-value of < 0.01 to indicate a statistically significant result.

A Spearman correlation was carried out to compare the length of the night with the weekly median total time in bedroom at night for both the cognitively intact group and the non-amnestic MCI group. For examining the correlation between time in bedroom and nighttime hours, the length of the night was calculated using:


(2) $$\begin{array}{rcl} N_{hr} & = & 24-D \end{array}$$

(3) $$\begin{array}{l} P=sin^{-1}(0.39795\;\cos(0.2163108\\ \;+2tan^{-1}(0.9671396\tan\;(0.00860(J-186))))) \end{array}$$

(4) $$\begin{array}{rcl} D & = & \left(\frac{24}{\pi }\right)\left(\frac{\sin\left(\frac{0.833\pi }{180}\right)+\sin\left(\frac{L\pi }{180}\right)\sin P}{\cos\frac{L\pi }{180}\cos P}\right) \end{array}$$

where D = hours of daylight, P = the revolution angle of the Earth, L = the latitude of Portland, Oregon (45.5231°N), and J = day of the year (for instance 1 for January 1) (32).

Change in seasonal temperature, obtained from the National Weather Service (33), over the course of the study period was also examined relative to rest-activity times by co-plotting of the mean daily temperature and the median total time in bedroom at night.

## Results

The cosinor fits are shown in Figures 1, 2. For the cognitively intact group, the MESOR is 7.41, the amplitude is 0.16, and the acrophase is −0.07. For the MCI group, the MESOR is 7.12, the amplitude is 0.04, and the acrophase is −4.58. The cosinor fit for the MCI group shows a much weaker relationship between total time in bedroom and day of year compared to the fit for the cognitively intact group.

The results of the rhythm detection test (the zero amplitude test) were significantly different by group. For the intact group, the *p*-value of the rhythm detection test was <0.001. For the non-amnestic MCI group, the *p*-value of the rhythm detection test was 0.3038. After adjusting for the known confounding of age, amplitude was not associated with MCI status (odds ratio = 1.167, 95% confidence = 0.69 to 1.92, *p*-value = 0.58).

**Figure 3:**
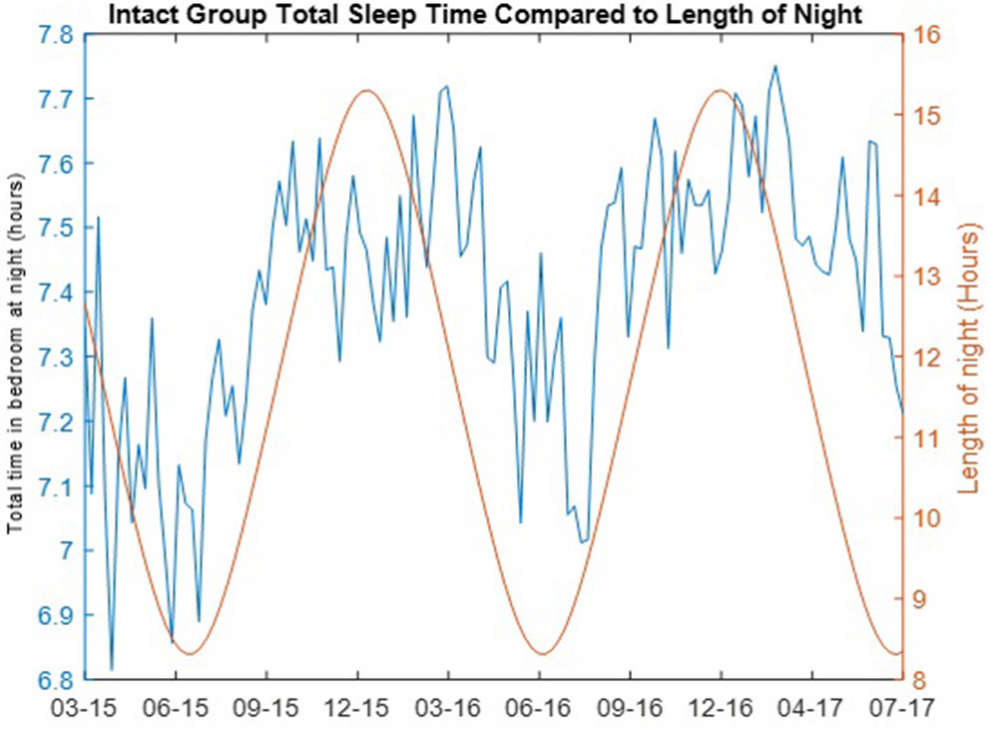
Total time in bedroom at night of cognitively intact group compared to length of night. The total time in bedroom at night in hours per night (blue) of the cognitively intact group per day of year is compared to the length of the night in hours (red) in Portland, Oregon over the length of the study.

**Figure 4:**
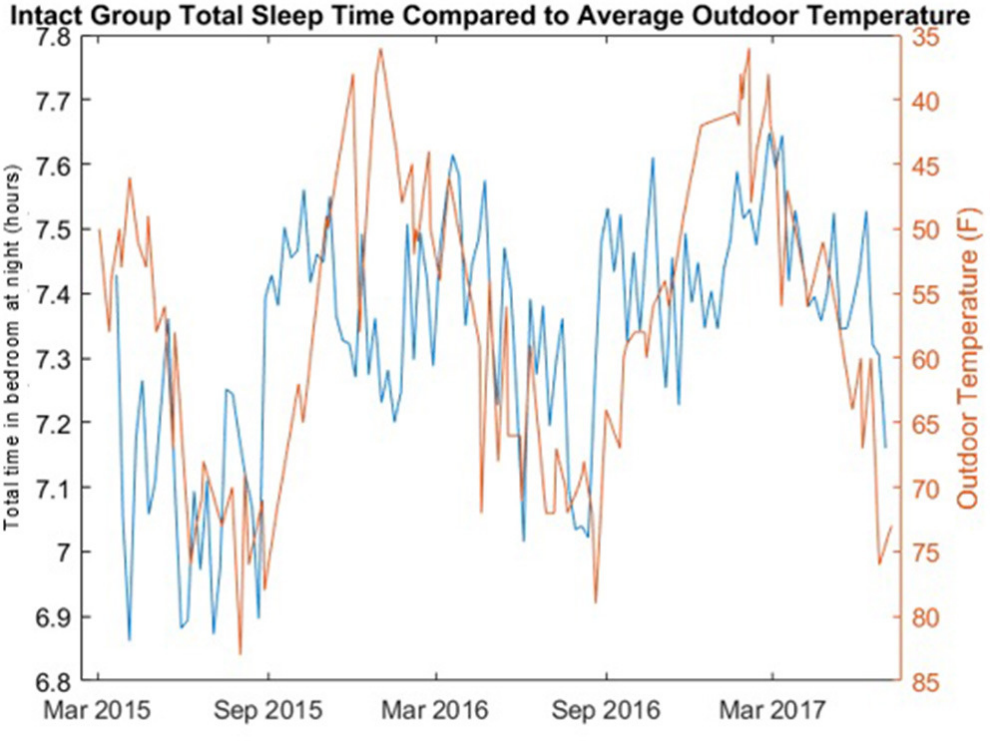
Total time in bedroom at night of cognitively intact group compared to average daily outdoor temperature. The total time bedroom at night in hours per night (blue) of the cognitively intact group per day of year is compared to the average daily outdoor temperature (red) in Portland, Oregon.

The correlations of the length of the night with the weekly median total time in bedroom for both the cognitively intact group and the non-amnestic MCI group was significantly different: Intact group, ρ = 0.5733 (*p* = 4.225e-122); non-amnestic MCI group, ρ = 0.0012 (*p* = 0.9897). The length of the night in hours for each day and the weekly median total time in bedroom for the cognitively intact group are plotted in Figure 3. A plot comparing the weekly median total time in bedroom of the intact group with the mean daily temperature is shown in Figure 4.

The cosinor analysis was then carried out for each individual, resulting in a value for amplitude, acrophase, and MESOR for each person's rest-activity data. A multivariate logistic regression was carried out with the cosinor amplitude as the output and age and MCI status as the predictors. In this multivariate logistic regression, age is associated with MCI status but amplitude is not.

## Discussion

We found that there appears to be a relationship between the time of year (season) and the total amount of time spent in the bedroom at night for cognitively intact older adults, but this relationship was not as strong for older adults with mild cognitive impairment. This relationship was weakest for older adults with non-amnestic MCI. We carried out two analyses to test for the existence of a rhythmic structure with a period of 1 year. For the cognitively intact group, the rhythm detection test was statistically significant for a fit of a cosine function with a period of 365.24 days. The correlation between the cognitively intact group's total rest times and the length of the night was also statistically significant. Conversely, for the non-amnestic MCI group, both tests failed to identify a statistically significant periodic structure in the data. This leads to two major conclusions. First, seasonal variability of sleep duration is preserved with advanced age implying maintenance of the sensitivity of internal infradian signal sensing and generating mechanisms in cognitively intact octogenarians. Second, in individuals with MCI, normal seasonal patterns of sleep duration are lost implying disruption of mechanisms serving to generate these rhythms.

The finding of a seasonal variation in total time in the bedroom at night for cognitively intact individuals aligns with previous studies showing longer sleep during winter. Our data spans multiple years of behavior patterns, which is a unique feature of our studies, which may capture changes in sleep behavior with aging which we are not addressing with this analysis. A study of healthy older Icelandic adults (mean age 79.7 ± 4.9 years) found men slept 25 min longer in the winter than in the summer (34) and a similar seasonal pattern was seen in healthy Danish children, who slept 12 min longer during the winter (9). An actigraphy study of medical students found total sleep time was 18 min greater in the autumn than the spring (35). Pre-industrial societies have also been reported to have slept longer during winter months based on actigraphy recorded in summer and winter months (8). Our study is among the first to compare seasonal rest-activity patterns continuously across the year in older adults who are cognitively intact and those who have MCI. Previously, we have shown using passive IR sensing of sleep behavior for over 26 weeks of nightly assessment that nightly sleep habits of individuals with MCI compared to cognitively intact older adults was more disturbed (5). Others using short-term (<10 nights) actigraphy recordings or surveys have also documented night-to-night sleep disruptions in MCI (18, 33, 36, 37).

The basis for these latter nightly disruptions are commonly attributed to pathologies accruing in brain systems crucial for maintaining core biological rhythms such as in the hypothalamus, suprachiasmatic nucleus, basal forebrain, and related systems of the brain (38). Among octogenarians, the pathologies that may populate these regions are likely to be heterogeneous including classic Alzheimer's pathologies (i.e., amyloid and tau protein aggregation, microvascular disease, and synapse loss). The basal forebrain has also been implicated in this clinical-pathological correlation (39, 40). Infradian rhythms are also mediated through these systems and likely result from the accumulation of these same pathologies.

The observation of disrupted seasonal rhythms in MCI may have important clinical implications. Although sleep disturbances appear more common in MCI and with frank dementia, it is not known whether these disturbances are perceived as being worse during winter months since this is the season when cognitively intact older adults diverged the most in terms of total sleep times compared to the MCI group. This insensitivity to seasonal change may affect the quality and severity of agitated behaviors and sleep disturbances in patients with more advanced dementia. Because of the differences in sleep duration depending on the season, seasonality needs to be considered during shorter term studies of sleep. The time of year when normative data is collected may matter; aggregating multi-subject data from 2 weeks of actigraphy recorded across a year may regress results to a mean that does not reflect true biological variability.

The strengths of our study include the long period of time over which participants were monitored, producing more than a year's worth of continuous data from passive sensors. Another strength is the fact that volunteers were monitored in their own homes rather than in an unfamiliar setting such as a sleep clinic or laboratory.

This study had several limitations. The main limitation is that we do not know if time spent motionless in the bedroom at night can be a proxy for total time asleep. The study of daytime sleep is also difficult to carry out using this technology because it is not possible to differentiate between sedentary behavior and sleep. The MCI group was made up of existing cases of MCI rather than incident cases, so we cannot say when these changes in sleep patterns begin, but the changes are likely early (all participants were living independently in the community) and are possibly a signal of future more severe cognitive decline. The sample was limited to a selected cohort of elderly; more diverse samples are needed. The cohort recruited from the ORCATECH Life Laboratory included a small number of participants with amnestic MCI, but the sample (9 individuals) did not have adequate available data to produce statistically valid results. Inspection of this data (not shown) suggests that there may also be disrupted infradian patterns, but additional study will be needed to make more definitive conclusions.

We also cannot generalize these results to other sub-types of MCI. Our study is also limited by our technology. Since the passive sensors do not directly measure sleep as in polysomnography, our rest-activity parameters are estimates inferred from observed night-time behavior. However, the accuracy of rest time calculated from the firing of these sensors was validated by comparing it with the sleep time detected by bed pressure mats placed under the bed. This approach was found to be comparable in quality with correlation coefficients of 0.99 for bed time and 0.96 for rising time (28).

The observations in this study suggest that changes in seasonal sleep patterns may be a sensitive early marker for mild cognitive impairment. Cognitive changes may be associated with a loss of sensitivity to environmental cues for sleep patterns. Future work would expand methodology of this study to include wrist actigraphy and bed mats to observe more seasonal cycles. Longitudinal studies of individuals over time would also be valuable to investigate how these sleep patterns change if an individual is diagnosed with mild cognitive impairment and then followed over the entire course of illness to brain autopsy. Studies of individuals living in different climates and latitudes would also be of great interest.

## Data Availability Statement

The original contributions presented in the study are included in the article/supplementary material, further inquiries can be directed to the corresponding author.

## Ethics Statement

The studies involving human participants were reviewed and approved by the Oregon Health and Science University Institutional Review Board (Life Laboratory, IRB #2765; ISAAC IRB #2353). The patients/participants provided their written informed consent to participate in this study.

## Author Contributions

CR and JK conceived of the presented analysis. CR developed the MATLAB code and performed the computations. NM carried out all statistical regressions. HD and ML verified the analytical methods. ZB supervised the collection of the data. JK supervised the findings of the work. All authors discussed the results and contributed to the final manuscript. All authors contributed to the article and approved the submitted version.

## Funding

This research described here was supported by grants from the National Institutes of Health, National Institute on Aging (P30 AG024978, P30 AG008017). ML received support from VA Career Development Award (CDA) # IK2 BX002712, VA CSRD Merit Review Award #I01 CX002022, Hartford Center for Gerontological Excellence, and Pacific Northwest-National Laboratory.

## Conflict of Interest

The authors declare that the research was conducted in the absence of any commercial or financial relationships that could be construed as a potential conflict of interest.

## Publisher's Note

All claims expressed in this article are solely those of the authors and do not necessarily represent those of their affiliated organizations, or those of the publisher, the editors and the reviewers. Any product that may be evaluated in this article, or claim that may be made by its manufacturer, is not guaranteed or endorsed by the publisher.
